# Deletion of the P/Q-Type Calcium Channel from Serotonergic Neurons Drives Male Aggression in Mice

**DOI:** 10.1523/JNEUROSCI.0204-22.2022

**Published:** 2022-08-24

**Authors:** Pauline Bohne, Achim Volkmann, Martin K. Schwarz, Melanie D. Mark

**Affiliations:** ^1^Behavioral Neuroscience, Ruhr-University Bochum, Bochum, D-44780, Germany; ^2^Institute of Experimental Epileptology and Cognition Research, University of Bonn Medical School, Bonn, D-53127, Germany

**Keywords:** aggression, Cav2.1, dorsal raphe, P/Q-type channel, serotonergic system, ventromedial hypothalamus

## Abstract

Aggressive behavior is one of the most conserved social interactions in nature and serves as a crucial evolutionary trait. Serotonin (5-HT) plays a key role in the regulation of our emotions, such as anxiety and aggression, but which molecules and mechanisms in the serotonergic system are involved in violent behavior are still unknown. In this study, we show that deletion of the P/Q-type calcium channel selectively from serotonergic neurons in the dorsal raphe nuclei (DRN) augments aggressive behavior in male mice, while anxiety is not affected. These mice demonstrated increased induction of the immediate early gene *c-fos* and *in vivo* serotonergic firing activity in the DRN. The ventrolateral part of the ventromedial hypothalamus is also a prominent region of the brain mediating aggression. We confirmed a monosynaptic projection from the DRN to the ventrolateral part of the ventromedial hypothalamus, and silencing these projections with an inhibitory designer receptor exclusively activated by a designer drug effectively reduced aggressive behavior. Overall, our findings show that deletion of the P/Q-type calcium channel from DRN neurons is sufficient to induce male aggression in mice and regulating its activity may serve as a therapeutic approach to treat violent behavior.

**SIGNIFICANCE STATEMENT** In this study, we show that P/Q-type calcium channel is mediating aggression in serotonergic neurons from the dorsal raphe nucleus via monosynaptic projections to the ventrolateral part of the ventromedial hypothalamus. More importantly, silencing these projections reduced aggressive behavior in mice and may serve as a therapeutic approach for treating aggression in humans.

## Introduction

Aggression and aggressive behavior are one of the most preserved social traits through nature, allowing the organism to defend itself, their offspring, resources, and their territory. In our modern society, aggressive behavior is considered destructive, unbeneficial, and often a consequence of not controlling one's impulses. This so-called impulsive aggression is mediated in part via the serotonergic system. 5-HT modulates our emotions, such as anxiety and aggression, as well as stress levels, but is also involved in cognition and memory. However, the exact regulatory mechanisms between 5-HT release and aggressive behavior are still not fully understood.

Various studies correlated insufficient 5-HT levels in the brain to increased aggression in humans (Coccaro, 1992; Coccaro et al., 1997; Stanley et al., 2000), monkeys (Zajicek et al., 2000), and rodents (Lucki, 1998; Ferrari et al., 2003) and tryptophan hydroxylase (TPH)-deficient mice were shown to be more aggressive than their control littermates; however, balancing out their 5-HT levels rescued the aggressive phenotype (Mosienko et al., 2012). Similarly, increasing 5-HT levels reduces aggression in animal models (Olivier et al., 1989) and patients (Barkan et al., 2006). Additionally, direct infusion of a 5-HT1_A_ receptor agonist into the dorsal raphe nuclei (DRN) of mice reduces the firing of serotonergic neurons and correlates with decreased aggressive behavior (De Boer et al., 2000; Caramaschi et al., 2007). Since the 5-HT1_A_ receptor serves as an inhibitory autoreceptor on DRN neurons (McDevitt and Neumaier, 2011), it is possible that autoregulatory mechanisms modulate DRN neuron firing, thus fine-tuning neuronal basis for aggression. Accordingly, we recently reported that overexpression of one of the regulators of G protein signaling (RGS), RGS2, in serotonergic neurons leads to aggression and enhanced firing of DRN neurons via facilitating GPCR-mediated inhibition of 5-HT1_A_ receptors on serotonergic neurons (Mark et al., 2019); however, it is not clear how synaptic transmission from serotonergic neurons onto targeted areas influences aggressive behavior in mice.

Synaptic transmission, neuronal excitability, and action potential generation all require calcium channels at the presynapse or soma to allow efficient signal transduction between neurons. The voltage-gated P/Q-type calcium channel (Ca_V_2.1), is preserved throughout the CNS in mammals, where it is expressed presynaptically and somatodendritically (Westenbroek et al., 1995). The channel is particularly involved in transmitter release as it is strongly coupled to exocytosis machinery (Qian and Noebels, 2001; Li et al., 2007). However, little is known about calcium channels in the DRN. One study verified that the Ca_v_2.1 is expressed on serotonergic neurons (Templin et al., 2012), and two additional studies showed that 5-HT has a regulatory effect on Ca_v_2.1 and N-type calcium channels (Bayliss et al., 1995, 1997a,b), suggesting a cooperative mechanism between 5-HT and calcium channels in altering aggressive behaviors.

In this paper, we aimed to investigate the influence of Ca_v_2.1 on serotonergic neurons and their involvement in regulating aggressive behavior using our *ePet-Cre/Cacna1a*^−/−^ mice, where the pore-forming α1A-subunit of the Ca_v_2.1 was deleted specifically from serotonergic neurons. We found that the Ca_v_2.1 deletion augmented aggressive, but not anxious, behavior in mice. This aggressive behavior directly correlated with increased serotonergic cell firing and *c-fos* expression in the DRN and ventrolateral part of the ventromedial hypothalamus (VHMvl). Additionally, we found that monosynaptic projections from the DRN to the VHMvl and their chemogenetic inhibition resulted in reduced aggressive behavior in *ePet-Cre/Cacna1a*^−/−^ mice, showing that the DRN directly influences aggressive behavior at the presynapse in the VHMvl.

## Materials and Methods

### Mice

*Cacna1a^Citrine^* mice (abbreviated *Cacna1a*^+/+^) (Mark et al., 2011) were bred with *ePet-Cre* mice (Scott et al., 2005) to obtain *ePet-Cre/Cacna*^−/−^ mice. *Gt(ROSA)26Sor^tm9(CAG-tdTomato)Hze^/J* mice (*tdTomato*, JAX #007909) (Madisen et al., 2010) were bred with *ePet-Cre/Cacna1a*^−/−^ mice to obtain mice expressing tdTomato under control of the ePet-1 enhancer (*ePet-Cre/Cacna1a*^−/−^*/tdTomato*). Transgene expression was detected by PCR analysis as follows: Cre forward: 5′-ATTCTCCCACCACCGTCAGTACG-3′, reverse: 5′- AAAATTTGCCTGCATTACCG-3′; Cacna1a forward: 5′-GGGGTCTGACTTCTGATGGA-3′, reverse: 5′-AAGTTGCACACAGGGCTTCT-3′; Cacna1a^Citrine^: forward 5′-TATATCATGGCCGACAAGCA-3′, reverse 5′-TTCGGTCTTCACAAGGAACC-3′; and tdTomato forward: 5′-GGCATTAAAGCAGCGTATCC-3′, reverse: 5′-CTGTTCCTGTACGGCATGG-3′. Male C57Bl/6J mice (JAX #000664) 8-12 weeks of age were used as intruder mice.

Mice were housed on a 12 h dark/light cycle with food and water *ad libitum*. The study was conducted in accordance with the European Communities Council Directive of 2010 (2010/63/EU) for care of laboratory animals and approved by the animal care committee of North Rhine-Westphalia, Germany, based at the LANUV (Landesamt für Umweltschutz, Naturschutz und Verbraucherschutz). The study was supervised by the animal welfare commission of the Ruhr-University Bochum. All efforts were made to minimize the number of mice used. All experiments were conducted during the wake cycle of the mice.

### Ethics approval

Keeping of experimental animals and all performed procedures were conducted with approval of a local ethics committee (Bezirksamt Arnsberg) and the animal care committee of North Rhine-Westphalia, Germany, based at the LANUV (LANUV; Landesamt für Umweltschutz, Naturschutz und Verbraucherschutz Nordrhein-Westfalen). The study was conducted in accordance with the European Communities Council Directive of 2010 (2010/63/EU) for care of laboratory animals and supervised by the animal welfare commission of the Ruhr-University Bochum.

### Histology

Mice were anesthetized with ketamine/xylazine (100/10 mg/kg, respectively) and transcardially perfused with ice-cold 4% PFA (Sigma-Aldrich) in 1× PBS, pH 7.4. Brains were dissected and postfixed for 1 h in 4% PFA in 1× PBS, then cryoprotected in 30% sucrose in 1× PBS and stored at 4°C. Brains were sliced using a cryostat (Leica, CM3050 S). For TPH staining, 30 µm free-floating sections were blocked in 10% NDS in 0.3% PBS-T for 1 h at room temperature, followed by incubation with primary antibody rabbit-anti-TPH2 (1:500, Abcam, #ab184505) in 3% NDS in 0.3% PBS-T overnight at 4°C. Secondary antibody donkey-anti-rabbit DyLight488 (Abcam, #ab96919) in 3% NDS in 0.3% PBS-T was added for 2 h at room temperature.

For *c-fos* staining, 35-µm-thick coronal sections from DRN and VHMvl were blocked in 4% FBS in 0.1% PBS-T for 1 h at room temperature. Primary antibody rabbit-anti-*c-fos* (1:1000, Santa Cruz Biotechnology) in blocking solution for 3 h at room temperature and then incubated in the secondary antibody goat-anti-rabbit DyLight 488 secondary antibody (1:1000, Invitrogen) in 10% FBS in 0.2% PBS-T overnight at 4°C. For staining of the glutamate transporter eAAC1, 25 µm sections from the VHMvl were blocked in 5% NDS in 0.3% PBS-T for 1 h at room temperature, followed by incubation of goat-anti eAAC1 (1:500, Merck) in blocking solution overnight at 4°C. The next day, donkey-anti-goat DyLight650 (1:500, Invitrogen, SA5-10 089) in blocking solution was added to the sections for 2 h at room temperature.

For staining of the Ca_v_2.1 (Mark et al., 2011), 20 µm sections of the DRN of *ePet-Cre/Cacna1a*^−/−^/*tdTomato* and *ePet-Cre/Cacna1a*^+/+^*/tdTomato* mice were blocked in 5% NDS in 0.3% PBS-T for 1 h at room temperature, incubated with primary antibody rabbit-anti-Ca_v_2.1 (1:500, SySy, #152203) in blocking solution overnight at 4°C. The secondary antibody donkey-anti-rabbit DyLight 650 (Invitrogen, #SA5-10 041) was incubated for 2 h at room temperature. Slices were mounted with Roti-Mount FluorCare (Roth) and dried at 4°C.

### Imaging

All images were acquired using a Leica TCS SP5 laser scanning microscope (Leica, DM16000 B, Wetzlar) and Leica Application Suite Advanced Fluorescence Software (LAS, AF 2.6). Multifluorescent images were obtained using the sequential scan mode. *z* stacks were made of each section, and crosstalk of fluorophores was eliminated automatically by the software. Images were further analyzed using ImageJ (National Institutes of Health).

### Behavior tests for aggression

#### Resident Intruder (RI) test

Three- to 6-month-old male *ePet-Cre/Cacna1a*^−/−^ mice were single housed in their cages for at least 14 d to allow development of territorial behavior. Cages were not cleaned during the duration of the test. Male intruder mice of 7-10 weeks of age were housed in groups. Resident mice were primed before the RI test until they showed aggressive behavior. For 9 consecutive days, one randomly chosen intruder mouse was placed into the resident's cage for 5 min. The latency to first attack and the number and the total duration of attacks (sum of duration of each attack) were noted, and the experiment video tracked using a Panasonic camcorder for later analysis. For the CNO experiments, mice were intraperitoneally injected with 5 mg/kg clozapine-*N*-oxide (CNO, HelloBio, HB6149, diluted in NaCl) for 3 consecutive days 30 min before testing, followed by 3 d of saline injection. Mice received a final dose of 5 mg/kg CNO and were killed for *c-fos* induction as described below.

#### Tube displacement test

A custom-made plastic tube (30 cm × 3 cm) was used for the tube displacement test. Two mice were placed into either end of the tube and given 5 min to push out their opponent. The first mouse to exit the tube was considered the “loser.” If both mice did not leave the tube, or exited the tube simultaneously, then it was considered a “tie.” All mice underwent 6 trials with randomized opponents. The tube was cleaned with 70% EtOH between trials. The number of winners per group is represented as percentage to the total trials performed. For the CNO experiments, mice were injected with 5 mg/kg CNO (i.p.) 30 min before testing.

#### Rotarod

The rotarod test (Columbus Instruments) was used to explore potential side effects on motor coordination and balance after intraperitoneal injection of CNO. Ten male *ePet-Cre/Cacna1a*^−/−^ mice were injected with 10 mg/kg CNO or saline 30 min before the test, then placed on the rod rotating at 4 rpm for 1 min for acclimation. The speed of the rod increased at 0.1 rpm/s up to 40 rpm until mice fell off the rod. The latency to fall and rpm were noted. Three trials per mouse were conducted and averaged.

### Behavior tests for anxiety

For characterizing the anxiety-like phenotypes of *ePet-Cre/Cacna1a*^−/−^ (7 female, 10 male) and *Cacna1a*^+/+^ (8 female, 10 male), mice at 3-6 months of age were tested according to previously published methods (Mark et al., 2019; Bohne et al., 2021). Mice were acclimated to the testing room 60 min before the tests.

#### Open Field Test

An opaque 50 × 50 cm Plexiglas chamber was subdivided into a center (20 × 20 cm), intermediate, and border (8 cm from walls) regions. The arena was brightly illuminated with 950 lux above the arena. Mice were placed into the center of the open field arena and video tracked for 15 min using the Ethovision XT 8.5 software (Noldus). To rule out possible side effects of CNO administration, *ePet-Cre/Cacna1a*^−/−^ mice were intraperitoneally injected with 10 mg/kg CNO or saline 30 min before the test. The total time spent in the center (s), latency to first enter the center (s), and total distance traveled (cm) were analyzed. The chamber was wiped with 70% EtOH between subjects.

#### Elevated Plus Maze

The test consisted of a maze of two 33 × 6 cm^2^ open arms, two 33 × 6 × 16.5 cm^2^ closed arms, and a 6 × 6 cm^2^ open center. The maze was elevated 43 cm above the floor. Mice were placed in one of the open arms and their explorative behavior video tracked using Ethovision XT 8.5 software (Noldus) for 5 min. The duration (s) and frequency (*n*) in open arms, the latency to first enter an open arm (s) and the number of head dips (*n*) were analyzed.

#### Place Preference Test

The Place Preference Test, also known as the light/dark preference test, was modified to Crawley and Goodwin (1980). An opaque Plexiglas arena (30 × 30 × 30 cm) was divided into two arenas. One open, light arena (950 Lux) and a closed arena darkened with black infrared see through Plexiglas with an opening connecting both chambers (30 × 15 × 30 cm). Mice were placed into the right corner of the light arena, and their explorative behavior was video tracked using Ethovision XT 8.5 software (Noldus) for 5 min. The time spent in the light zone (s) and latency to first transit serve as an indicator for anxious behavior. The chambers were rinsed with 70% EtOH between subjects. Each mouse underwent one trial.

#### Novelty Suppressed Feeding

Mice were food-deprived for 24 h but had access to water *ad libitum* before testing. A familiar food pellet previously weighted (∼2 g) was placed in the center of a brightly illuminated, aversive arena (30 × 30 × 30 cm). Mice were placed into a plastic tube in the right corner of the arena, which was removed from at the beginning of the test. Mice were video tracked using the Ethovision XT 8.5 software (Noldus) for a maximum of 10 min. The task ended, when mice first fed, defined as biting the food pellet and holding it with their forepaws. The latency to start feeding was an indicator for anxious behavior. Mice were allowed to consume food for an additional 5 min within the arena. The mouse was then moved to its home cage. The food pellet was weighted to analyze food consumption between groups. Each mouse underwent one trial.

### Extracellular *in vivo* recordings of serotonergic DRN neurons

Recordings from serotonergic neurons in the DRN of male *ePet-Cre/Cacna1a*^−/−^ and *Cacna1a*^+/+^ mice were made as previously described (Kruse et al., 2014; Mark et al., 2019) using a 7 channel multielectrode matrix “Eckhorn microdrive” (Thomas Recording). Animals were deeply anesthetized with 1.2%-2% isoflurane and placed in a stereotaxic frame (Narishige). A craniotomy of 2 mm × 2 mm size above the DRN (AP: −4.6 mm) was performed. The exposed brain was kept moist with ACSF and the dura mater remained intact; 2 mΩ, 80-µm-diameter quartz electrodes (Thomas Recording) were used for single-cell recordings at DV: −3.2 to 2.7 mm. Signals were amplified and filtered (bandpass, 0.1-8 kHz) with a multichannel signal conditioner (CyerAmp380, Molecular Devices) and sampled with 32 kHz via a A/D converter (NI PCI-6259 multifunction data acquisition board, National Instruments), controlled via a custom-made software using MATLAB (The MathWorks) as previously published (Kruse et al., 2014). Recordings were saved externally and stored for offline analysis using a custom-written MATLAB (The MathWorks) program (Kruse et al., 2014). Potential serotonergic neurons were identified by their typical broad spikes and slow, regular firing (∼2 Hz) (Aghajanian et al., 1978; Vandermaelen and Aghajanian, 1983; Mark et al., 2019). According to Beck and Kirby (Kirby et al., 2003; Beck et al., 2004), error rates using AP duration as a criteria to distinguish serotonergic from nonserotonergic neurons in the DRN are ∼20%-40%.

Firing rates, amplitude of spikes, and coefficients of variation (CVs) of interspike intervals (ISIs, CV1 and CV2) were analyzed and compared between the two groups. To quantify the spike train regularity, CVs of ISIs were calculated. Additionally, the CVs for adjacent intervals, CV2 ([2(Δti + 1 − Δti)] (Δti + 1 + Δti)−1), of ISIs were calculated (Mark et al., 2015). An average of CV2 over *n* estimates the intrinsic variability of a spike train, nearly independent of slow variations in average rate. All statistical analyses were calculated by means of one-way ANOVA. Results are presented as mean ± SEM. Exponential fit of activity after stimulation onset and offset was made with IgorPro (WaveMetrics). Recordings were performed from *n* ≥ 6 mice/group.

### *c-fos* induction studies

Three groups of 4- to 6-month-old, male *ePet-Cre/Cacna1a*^−/−^ and *Cacna1a*^+/+^ mice (each *n* = 3/group) underwent the RI test as previously described but were perfused on day 0 (RI 0), day 3 (RI 3), or day 7 (RI 7) 90 min after exposure to the intruder to allow expression of the immediate early gene *c-fos*. Immunohistochemistry was conducted as described above.

### Virus production and intracranial injections

Recombinant adeno-associated viruses, SADΔG-eGFP (EnVA) and helper plasmids were produced as already described (Niedworok et al., 2012; Bohne et al., 2019). The inhibitory designer receptor exclusively activated by a designer drug (DREADD) receptor hM4D(Gi) was produced as AAV9-EF1α-DIO-hM4D(Gi)-mCherry and injected into the DRN at AP: −4.4 mm, ML: 0 mm, and DV: −3.3 to 2.7 mm of *ePet-Cre/Cacna1a*^−/−^ mice (*n* = 5). The corresponding plasmid was created by amplification of hM4D(Gi)-mCherry from an C vector (#50475, Addgene) and inserted into an AAV-EF1α vector. AAV8-CMV-Cre (Bohne et al., 2019) was injected into the DRN of tdTomato mice (*n* = 2). Helper viruses AAV2-CBA-mRFP-IRES-TVA and AAV2-CBA-RG were mixed in a 1:1 ratio and injected into the VHMvl at AP: −1.5 mm, ML: 0.6 mm, DV: −5.3 mm of *ePet-Cre/Cacna1a*^−/−^ and *ePet-Cre/Cacna1a*^+/+^ mice (*n* = 6/group) 1 week before injection of modified rabies viruses SADΔG-eGFP (EnVA). Intracranial injections were performed as previously described (Bohne et al., 2019). Briefly, mice received 2 mg/kg carprofen subcutaneously 30 min before surgery. Animals were deeply anesthetized with 1.2%-2% isoflurane (cp-Pharma) and placed into a stereotaxic frame (Narishige). The skin above the skull was opened with a small incision, and a craniotomy was drilled through the skull above the injection side; 0.2 µl of virus was manually inserted into a glass pipette of 0.2 µm diameter, connected to a 5 ml syringe to allow pressure injection of the virus in 50-100 µm intervals. A total of 2 min was given between intervals to allow diffusion of the virus into the tissue. The wound was sutured (Surgicryl fMonofilament), and mice were removed to their home cages. Animals were closely monitored and received 2 × 2 mg/kg carprofen daily for the next 3 d. Mice were given 4 weeks to recover for RI experiments. For tracing experiments involving modified rabies viruses, mice were perfused 1 week after tracer virus was injected.

### Statistics and reproducibility

Statistical analysis was performed using SigmaPlot 12.5 (Systat Software). Data were analyzed for normality by Shapiro–Wilk test (*p* ≥ 0.05) and equal variance tested with equal variance test (*p* ≥ 0.05). In case both tests were passed, a *t* test for comparison of two groups was conducted. If the tests were not passed, a Mann–Whitney Rank Sum test was conducted for comparison of two groups. Statistical significance was evaluated with a two-way repeated measures ANOVA for comparison of 2 groups of animals used for 2 or more different conditions. Data are mean ± SEM. *c-fos* counts were conducted manually in *n* ≥ 3 mice using ImageJ (National Institutes of Health). The *n* for every experiment is reported in the figure legends. Counts for ePet promoter efficiency were conducted manually using ImageJ (National Institutes of Health) in *n* ≥ 6 mice, separating the DRN into ventrolateral part of the dorsal raphe (DRVL), dorsal part of the raphe nucleus (DRD), and ventral part of the dorsal raphe nucleus (DRV) (comprising DRI, DRV, and DRc).

### Data availability statement

All materials and data supporting this study are available from the corresponding author on reasonable request.

## Results

### Generation of transgenic *ePet-Cre/Cacna1a*^−/−^ mice

**Figure 1.**
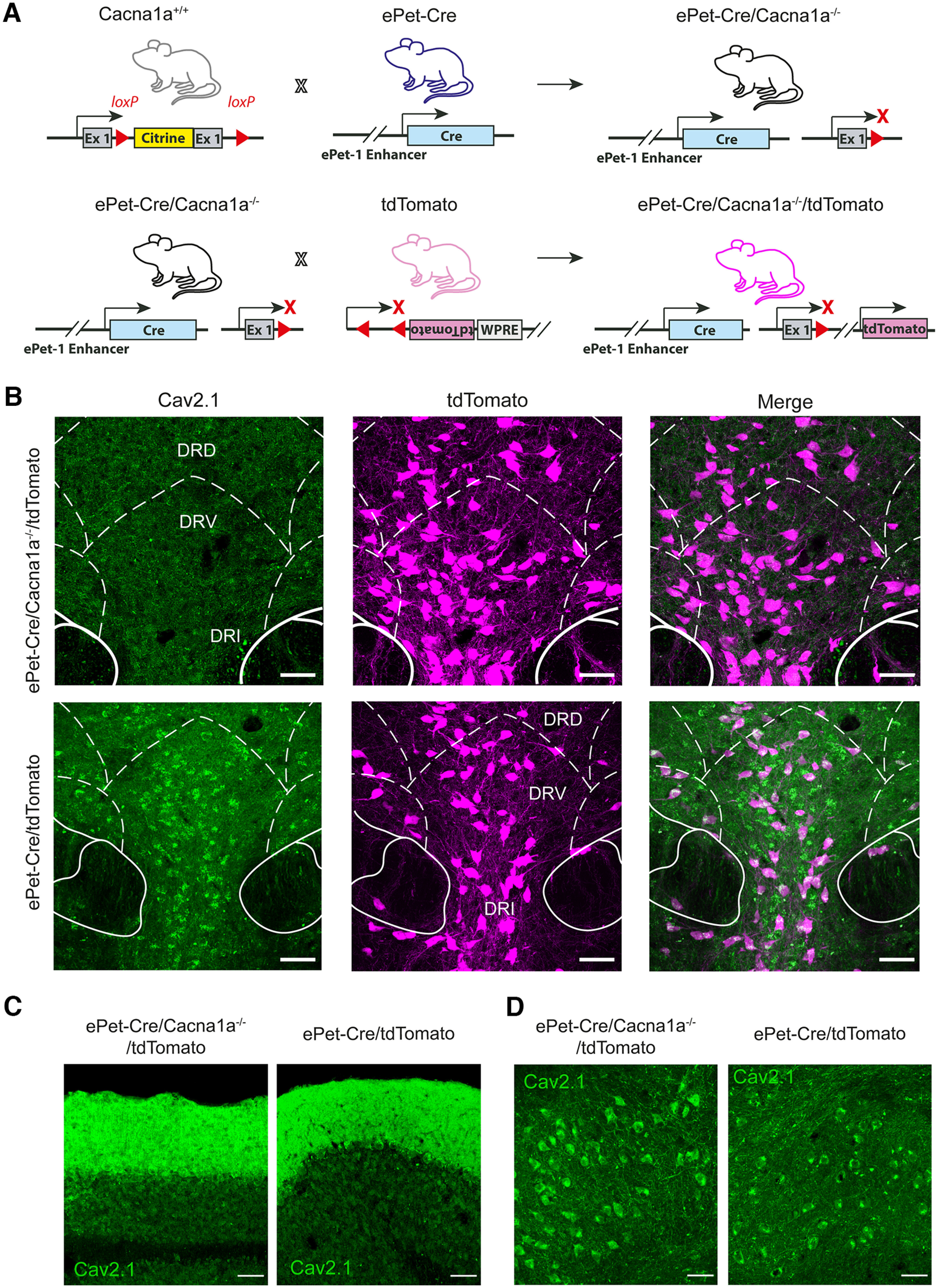
Creation of transgenic *ePet-Cre/Cacna1a*^−/−^ mice results in robust Cre expression and deletion of the Ca_v_2.1 from serotonergic DRN neurons. ***A***, *Cacna1a*^+/+^ mice were engineered with loxP sites flanking exon 1 (Ex 1) of the Ca_v_2.1 α1A subunit, known as *Cacna1a*. *Cacna1a*^+/+^ mice were crossed with *ePet-Cre* mice, which express Cre recombinase under control of the ePet-1 enhancer. This pairing results in *ePet-Cre/Cacna1a*^−/−^ offspring, where the Ca_v_2.1 is selectively removed from serotonergic neurons. To additionally enable intrinsic labeling of serotonergic neurons, *ePet-Cre/Cacna1a*^−/−^ offspring were crossed with tdTomato mice, resulting in *ePet-Cre/Cacna1a*^−/−^*/tdTomato* mice where Cre-dependent tdTomato (magenta) is selectively expressed in serotonergic neurons. ***B***, Example images of the DRN from a homozygous *ePet-Cre/Cacna1a*^−/−^*/tdTomato* (top) and control *ePet-Cre/tdTomato* mouse (bottom) depicting serotonergic neurons (magenta). As expected, there was no staining of Ca_V_2.1 (green) in the DRN serotonergic neurons of *ePet-Cre/Cacna1a*^−/−^*/tdTomato* mice, while the channel is still expressed in control mice. As a control, staining against the Ca_V_2.1 channel in cerebellar Purkinje and granule cells (***C***) as well as in deep cerebellar nuclei (DCN) neurons (***D***), was performed to verify that the channel was not deleted from other brain areas in both control *Cacna1a*^+/+^ and *ePet-Cre/Cacna1a*^−/−^ mice. Scale bars, 50 µm. exon 1, Ex 1; woodchuck hepatitis virus posttranslational regulatory element, WPRE; dorsal part of the raphe nucleus, DRD; ventral part of the raphe nucleus, DRV; interfasicular part of the raphe nucleus, DRI.

**Figure 2.**
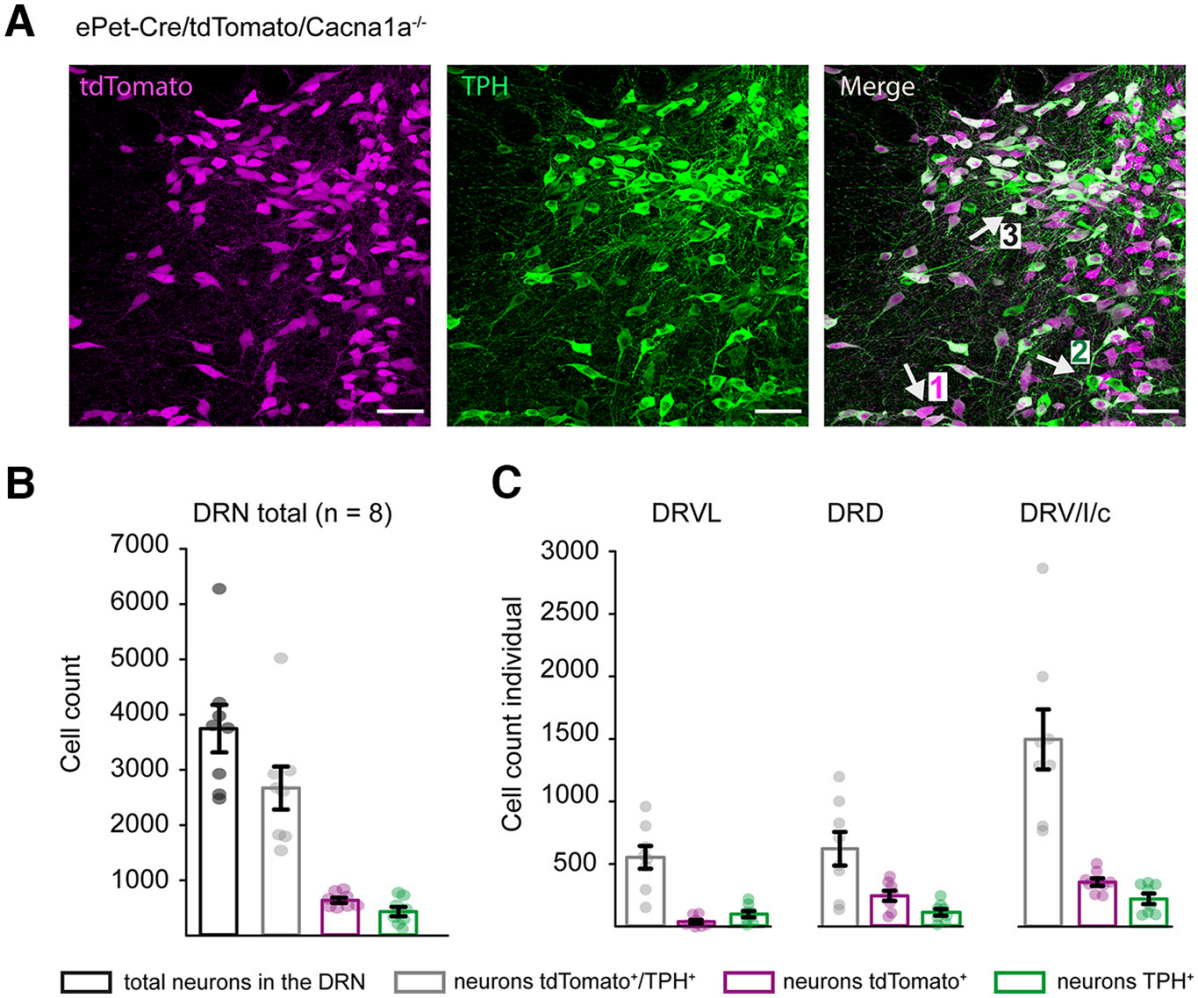
Characterization of the effectiveness of the serotonergic ePet-1 enhancer. ***A***, Example images of the DRN of an *ePet-Cre/Cacna1a*^−/−^*/tdTomato* mouse expressing tdTomato (magenta) under control of the ePet-1 enhancer. TPH staining (green) was used as a marker to identify serotonergic neurons. The merge of both channels (right site) allows identification of neurons, which are tdTomato^+^ (1), tryptophan hydroxylase (TPH^+^) (2), or positive for both (3). Scale bars, 50 µm. ***B***, The DRN of *ePet-Cre/Cacna1a*^−/−^*/tdTomato* mice was analyzed for neurons, which expressed both tdTomato and TPH (gray, 69.98 ± 6.98%), neurons only expressing tdTomato, which are not serotonergic (magenta, 18.77 ± 7.81%) or neurons, which express TPH but not tdTomato (green, 11.25 ± 4.96%). The number of cells analyzed in the DRN is reported as total cell count (black, 3747 ± 430 cells). Data are mean ± SEM. ***C***, The DRN cell count was separated into the ventrolateral part of the dorsal raphe nucleus (DRVL), dorsal part of dorsal raphe nucleus (DRD), and ventral part of dorsal raphe nucleus (DRV) combined with caudal (DRc) and interfascicular parts (DRI). Data are mean ± SEM. A total of 8 mice was analyzed.

The involvement of the serotonergic system in emotions, such as aggression and anxiety, in both humans and mice has long been reported (Linnoila et al., 1983; Chiavegatto et al., 2001; Audero et al., 2013; Nordman and Li, 2020). Ca_V_2.1 is expressed on serotonergic neurons in the DRN and is modulated by 5-HT (Bayliss et al., 1995, 1997a,b; Templin et al., 2012). However, its function and contribution to aggressive behavior have not been investigated. To determine whether the Ca_v_2.1 is involved in the regulation of aggressive behavior in the DRN, we selectively deleted the channel from serotonergic neurons in the DRN by crossing *ePet-Cre* mice expressing Cre recombinase under control of the ePet-1 enhancer (Scott et al., 2005) with our *Cacna1a*^+/+^ mice, where two loxP sites are located within exon 1 and intron 1 of the pore-forming α1A subunit gene of the Ca_v_2.1 (Mark et al., 2011). We generated *ePet-Cre/Cacna1a*^−/−^ mice, where the calcium channel is selectively deleted from serotonergic neurons in the DRN (Fig. 1*A*). We confirmed the deletion by immunohistochemical staining (Bomben et al., 2016; Mark et al., 2019) and found no expression of the Ca_v_2.1 in DRN serotonergic neurons of *ePet-Cre/Cacna1a*^−/−^ mice compared with *Cacna1a*^+/+^ mice (Fig. 1*B*). As expected, the Ca_v_2.1 was abundantly expressed in Purkinje and granule cells of the cerebellum and deep cerebellar nuclei (Fig. 1*C*,*D*). To examine the efficiency of the ePet-1 enhancer, we generated an *ePet-Cre/Cacna1a*^−/−^*/tdTomato* mouse line, where tdTomato expression was under control of the ePet-1 enhancer and compared the number of tdTomato positive (^+^) neurons, which colocalized with the serotonergic specific marker TPH (Spoida et al., 2014). The murine DRN was shown to contain ∼9000 serotonergic neurons (Ishimura et al., 1988), resulting in 5-HT as its main neurotransmitter; however, other neurotransmitters are present in the DRN as well, including glutamate, GABA, and dopamine (Luo et al., 2015). We found that 69.98 ± 6.98% of TPH^+^ neurons in the DRN colocalized with tdTomato^+^ neurons in *ePet-Cre/Cacna1a*^−/−^*/tdTomato* mice (Fig. 2*A*,*B*). The best efficiency of tdTomato/TPH^+^ cells was observed in the DRVL with 79.39 ± 6.5%, followed by ventral, interfascicular, and caudal parts (DRV/I/c) with 70.56 ± 7.04%. The DRD showed 60.97 ± 9.34% tdTomato/TPH^+^ cells (Fig. 2*C*). Together, the data indicate that the majority of the Ca_v_2.1 was selectively deleted from serotonergic neurons in the DRN.

### Ca_v_2.1 removal from serotonergic neurons leads to male aggression, but not increased anxiety-like behaviors

**Figure 3.**
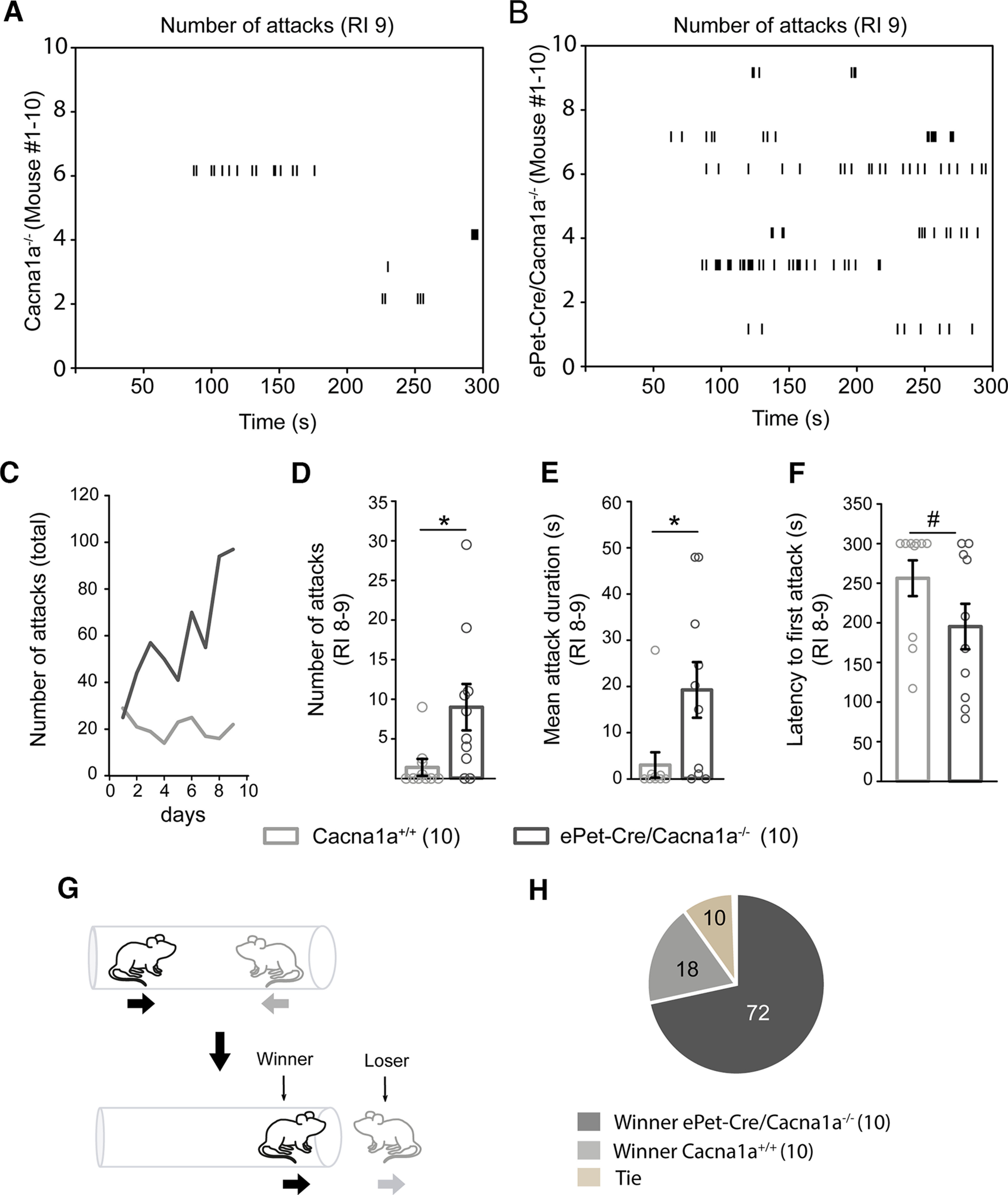
*ePet-Cre/Cacna1a*^−/−^ mice show increased aggressive behavior in the RI and displacement test. During the RI test, *Cacna1a*^+/+^ mice (***A***) attacked the intruder less often than *ePet-Cre/Cacna1a*^−/−^ mice (***B***) over a 10 min testing period on RI day 9 (RI 9). ***C***, *ePet-Cre/Cacna1a*^−/−^ mice showed an escalation in aggression during the RI test compared with control Cacna1a^+/+^ mice as demonstrated by an increase in the total number of attacks. The average number of attacks (*p* = 0.015) (***D***) and mean attack duration (*p* = 0.017) (***E***) on RI 8-9 was also augmented in *ePet-Cre/Cacna1a*^−/−^ compared with *Cacna1a*^+/+^ mice. ***F***, *ePet-Cre/Cacna1a*^−/−^ mice displayed a tendency for a reduced latency to first attack compared with control mice (*p* = 0.061). ***G***, Schematic for the displacement test. Two mice are placed into a plastic tube and given 5 min to push the opponent out of the tube. The mouse leaving the tube first is considered the “loser,” the other mouse the “winner.” ***H***, *ePet-Cre/Cacna1a*^−/−^ mice won 72% of their battles against their control opponents, while only 18% of the battles were won by *Cacna1a*^+/+^ in the displacement test. Ten percent of the trials ended in a tie. Six trials/mouse were performed with random opponents. The number of mice per group is indicated in parentheses. ***C-F***, Data are mean ± SEM. Statistical significance was evaluated with Mann–Whitney Rank Sum test (*p < 0.05; ^#^*p* = 0.061). See also Table 1.

Next, we characterized our *ePet-Cre/Cacna1a*^−/−^ and control *Cacna1a*^+/+^ mice for alterations in anxiety-like and male aggressive behavior. Ca_V_2.1-deficient *ePet-Cre/Cacna1a*^−/−^ mice in serotonergic neurons exhibited male aggression with no differences in their anxiousness compared with control mice in the open field, elevated plus maze, place preference and novelty suppressed feeding tests (Table 1). Male *ePet-Cre/Cacna1a*^−/−^ mice demonstrated increasing total number of attacks (605 attacks) compared with male *Cacna1a*^+/+^ mice (230 attacks, Fig. 3*C*) in the RI test. Peak aggression levels were observed on day 8-9 of the RI test for both Ca_V_2.1 mutant lines (Fig. 3*A*,*B*; Movies 1, 2), where *ePet-Cre/Cacna1a*^−/−^ mice showed a significant increase in aggressive behavior compared with control mice (9.0 ± 2.9 attacks vs 1.4 ± 0.89 attacks, *T* = 136.5, *p* = 0.015, Mann–Whitney *U* test), represented by the mean number of attacks (Fig. 3*D*). Additionally, the total attack duration of *ePet-Cre/Cacna1a*^−/−^ mice was significantly longer than control mice (19.24 ± 6.01 s vs 3.01 ± 2.75 s; *T* = 136, *p* = 0.017, Mann–Whitney *U* test) (Fig. 3*E*) and their latency to first attack was reduced (*ePet-Cre/Cacna1a*^−/−^ 195.3 ± 28.69 s to *Cacna1a*^+/+^ 256.3 ± 22.60 s, *T* = 80.5, *p* = 0.061, Mann–Whitney *U* test) (Fig. 3*F*). During the tube displacement test, an *ePet-Cre/Cacna1a*^−/−^ mouse and a control littermate were placed at one side of a plastic tube and the mouse exiting the tube first was considered “loser” (Fig. 3*G*). Not surprisingly, 72% of *ePet-Cre/Cacna1a*^−/−^ mice won against littermate controls in a total of 60 trials with randomized opponents, supporting the RI results that removal of the Ca_v_2.1 from the serotonergic neurons lead to aggressive behavior. Ten percent of trials were ties, and only 18% of trials were won by control *Cacna1a*^+/+^ mice (Fig. 3*H*; Movie 3). Together, our data suggest that Ca_v_2.1 deletion from serotonergic DRN neurons results in enhanced aggressive behavior, with negligible effects on anxiety-like behavior.

**Table 1.** Anxiety tests in *Cacna1a*^+/+^ and *ePet-Cre/Cacna1a*^−/−^ mice*^a^*

Parameter	Cacna1a^+/+^	ePet-Cre/Cacna1a^−/−^	*p*
Open field			
Duration in center (s)	174.80 ± 18.69	129.87 ± 9.54	0.062
Latency to first (s)	18.56 ± 4.43	15.81 ± 5.19	0.456
Elevated Plus Maze			
Duration in open arms (s)	8.73 ± 2.64	14.96 ± 4.16	0.256
Frequency in open arms (s)	1.11 ± 0.32	1.59 ± 0.33	0.251
Latency to first (s)	210 ± 29.54	133.29 ± 30.40	0.054
Number of head dips	1.22 ± 0.42	2.29 ± 0.78	0.465
Place Preference Test			
Duration in light zone (s)	78.86 ± 9.03	93.06 ± 5.20	0.166
Latency to first (s)	37.79 ± 21.27	11.18 ± 3.27	0.852
Novelty Suppressed Feeding			
Latency to eat (s)	116.89 ± 22.87	65.53 ± 10.13	0.102
Food consumption (mg)	104.0 ± 11.70	139.0 ± 10.50	0.032

*^a^*Data are mean ± SEM.

Movie 1. Example video of an *ePet-Cre/Cacna1a*^−/−^ mouse during the RI test. A foreign, male intruder was placed into the cage of a resident *ePet-Cre/Cacna1a*^−/−^ mouse for the RI test. The resident mouse attacked the male intruder with a shorter latency to first attack and displayed more attacks, while the intruder was submissive. 10.1523/JNEUROSCI.0204-22.2022.video.1

Movie 2. Example video of a control *Cacna1a*^+/+^ mouse during the RI test. A foreign intruder was placed into the cage of a resident *Cacna1a*^+/+^ mouse for the RI test. The *Cacna1a*^+/+^ mouse displayed less aggressive behavior toward the intruder. Instead, the resident showed curious behavior and social interest toward the intruder, such as sniffing and cleaning. 10.1523/JNEUROSCI.0204-22.2022.video.2

Movie 3. Example video of the tube displacement test. An *ePet-Cre/Cacna1*^−/−^ mouse (right) and a *Cacna1a*^+/+^ mouse (left) were placed in either end of a tube. The *Cacna1a*^+/+^ mouse exited the tube first and was considered the “loser.” 10.1523/JNEUROSCI.0204-22.2022.video.3

### Ca_v_2.1 deletion from serotonergic neurons results in increased neuronal activity in DRN and VHMvl in aggressive *ePet-Cre/Cacna1a*^−/−^ mice

**Figure 4.**
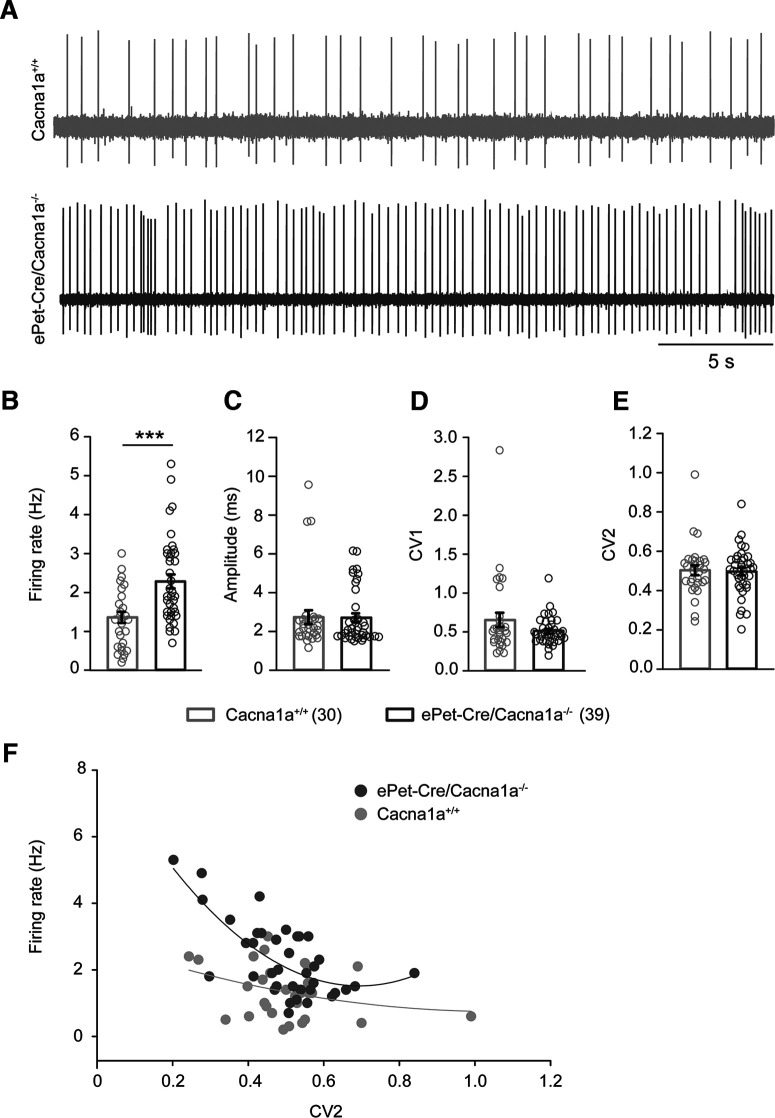
Electrophysiological characterization of putative DRN serotonergic neurons in *ePet-Cre/Cacna1a*^−/−^ mice reveals increased firing rates. ***A***, Example traces of neurons in the DRN of control *Cacna1a*^+/+^ and *ePet-Cre/Cacna1a*^−/−^ mice. Recordings were performed extracellularly in anesthetized mice. Calibration: 5 s. ***B***, Offline analysis of recordings from potential serotonergic neurons revealed an enhanced mean firing frequency in *ePet-Cre/Cacna1a*^−/−^ mice (bottom, dark gray, 2.284 ± 1.105 Hz, *n* = 39) compared with controls (top, light gray, 1.360 ± 0.776 Hz, *n* = 30). ***C***, The amplitude of recorded putative serotonergic neurons, as well as both CV1 (*Cacna1a*^+/+^ 0.62 ± 0.09, *ePet-Cre/Cacna1a*^−/−^ 0.511 ± 0.0281) (***D***) and CV2 (*Cacna1a*^+/+^ 0.503 ± 0.025, *ePet-Cre/Cacna1a*^−/−^ 0.121 ± 0.02) (***E***) did not differ between *Cacna1a*^+/+^ and *ePet-Cre/Cacna1a*^−/−^ mice. ***F***, Increased firing rates did not correlate with increased irregular firing. Recordings were performed in 8 control *Cacana1a*^+/+^ and 15 *ePet-Cre/Cacna1a*^−/−^ mice, respectively. Data are mean ± SEM. ***p < 0.001 (Mann–Whitney Rank Sum test). CV, coefficient of variation.

Alterations of 5-HT receptors or 5-HT levels were shown to induce aggressive behavior and result in altered cell firing (Olivier, 2004; Coccaro et al., 2015; Mark et al., 2019; Gorlova et al., 2020). Since Ca_V_2.1 is required for normal synaptic transmission, we investigated the physiological impact of Ca_v_2.1 deletion from serotonergic neurons by i*n vivo* extracellular recordings (Fig. 4). Using a multielectrode system, we recorded spontaneously active, putative serotonergic neurons in the DRN via identification by their typical broad spikes and slow, regular firing (∼2 Hz) (Aghajanian et al., 1978; Vandermaelen and Aghajanian, 1983; Mark et al., 2019). We found a higher firing frequency in *ePet-Cre/Cacna1a*^−/−^ compared with *Cacna1a*^+/+^ mice (2.28 ± 0.17 Hz vs 1.36 ± 0.14 Hz, *T* = 751, *n*(small) = 30, *n*(big) = 38, *p* ≤ 0.001, Mann–Whitney *U* test) (Fig. 4*A*,*B*). However, no alterations in the amplitude (Fig. 4*C*, 2.71 ± 0.23 ms to 2.74 ± 0.36 ms, *p* = 0.721, Mann–Whitney *U* test) were observed. To quantify the spike train regularity, we analyzed the CVs for ISIs. No differences between ISIs CV1 (Fig. 4*D*, 0.51 ± 0.02 to 0.65 ± 0.09, *p* = 0.659, Mann–Whitney *U* test) or adjacent intervals CV2 (Fig. 4*E*, 0.49 ± 0.02 to 0.50 ± 0.02, *p* = 0.838, Mann–Whitney *U* test) between mouse lines were observed. Although DRN neurons in the *Ca_V_2.1 ePet-Cre/Cacna1a*^−/−^-deficient mice fire significantly faster compared with controls, it does not correlate to higher irregular firing of cells (Fig. 4*F*).

**Figure 5.**
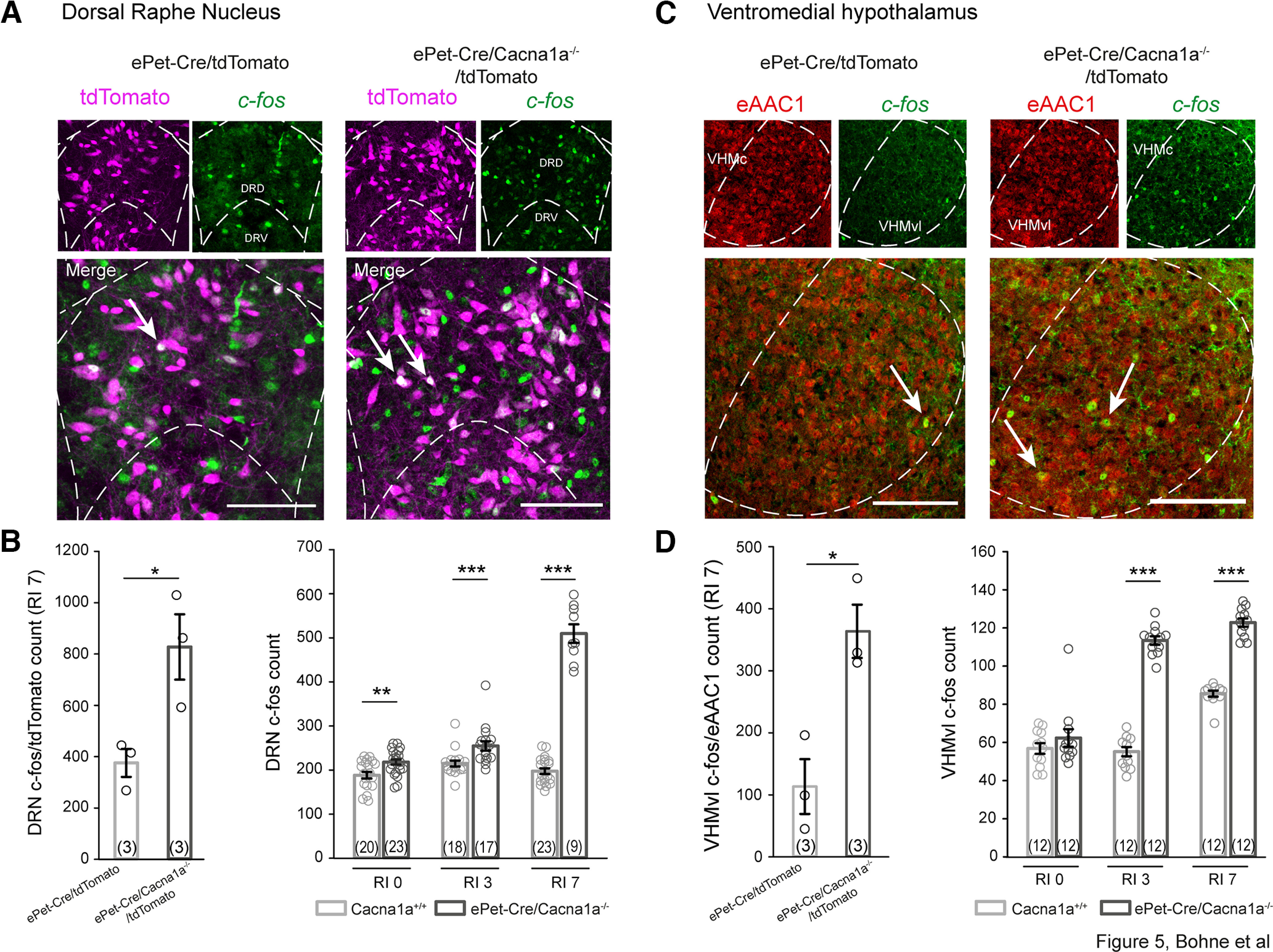
Aggressive behavior is correlated to the immediate early gene *c-fos* expression in both DRN and VHMvl of male *ePet-Cre/Cacna1a*^−/−^ mice. ***A***, Immediate early gene marker *c-fos* (green) induction in the DRN after 7 d of RI tests from *ePet-Cre/Cacna1a*^−/−^*/tdTomato* and *ePet-Cre/tdTomato* mice. Scale bars, 100 µm. ***B***, Quantitative analysis of these mice reveals significantly more *c-fos*/serotonergic-positive cells in the DRN in *ePet-Cre/Cacna1a*^−/−^*/tdTomato* (dark gray) compared with *ePet-Cre/tdTomato* control (light gray) mice (*n* = 3, *p* = 0.034). Additional analysis was conducted in control *Cacna1a*^+/+^ mice (light gray) and *ePet-Cre/Cacna1a*^−/−^ (dark gray) after 0, 3, and 7 d of RI tests. On all test days, *ePet-Cre/Cacna1a*^−/−^ showed higher neuronal activity in the DRN as indicated by the increased number of *c-fos*-positive cells compared with *Cacna1a*^+/+^ mice (RI 0 *p* = 0.007, RI 3 *p* ≤ 0.001, RI 7 *p* ≤ 0.001). ***C***, Seven days of RI induce c-fos expression (green) in glutamatergic (eAAC1, red) neurons in the VHMvl. Scale bars, 100 µm. ***D***, Mice presented in ***B*** were also analyzed for *c-fos* expression in the VHMvl. Comparison of *ePet-Cre/Cacna1a*^−/−^*/tdTomato* (dark gray) and *ePet-Cre/tdTomato* control (light gray) mice reveals significantly more *c-fos*/eAAC1 cells in *ePet-Cre/Cacna1a*^−/−^*/tdTomato* mice (*n* = 3, *p* = 0.008). The number of *c-fos*-positive cells in the VHMvl of ePet-Cre/Cacna1a^−/−^ mice showed no difference at RI 0 (*p* = 0.544) but increases at RI 3 (*p* ≤ 0.001) and RI 7 (*p* ≤ 0.001). Three mice/group were tested. The number of slices analyzed is given in parentheses. Data are mean ± SEM. VHMc, Central part of the ventromedial hypothalamus. Statistical significance was evaluated with *t* test for VHMvl counts or Mann–Whitney Rank Sum test (for DRN counts: **p < 0.01; ***p < 0.001).

We were also able to confirm an increase in DRN neuronal activity by *c-fos* induction in correlation with rising aggressive behavior in our *ePet-Cre/Cacna1a*^−/−^ and *ePet-Cre/Cacna1a*^−/−^/*tdTomato* mice. Initial analysis of *c-fos* expression after 7 d of the RI test (RI 7) (Fig. 5*A*) revealed significantly more *c-fos* in serotonergic neurons in *ePet-Cre/Cacna1a*^−/−^*/tdTomato* mice (dark gray) compared with *ePet-Cre/tdTomato* mice (light gray) (827.667 ± 127.314 vs 375.667 ± 54.48 cells, *t*_(4)_ = −3.264, *p* = 0.031, *t* test) (Fig. 5*B*). *c-fos* levels in the DRN were thus further monitored after 0, 3, and 7 d of the RI test (RI 0, RI 3, RI 7; Fig. 5*B*) in *ePet-Cre/Cacna1a*^−/−^ and littermate controls. In agreement with their increased serotonergic firing rates, *ePet-Cre/Cacna1a*^−/−^ mice displayed significantly more *c-fos* in the DRN at all time points examined compared with their control littermates (RI 0: 218.21 ± 5.86 to 214.94 ± 6.77, *T* = 325.5, *p* = 0.007; RI 3: 254.94 ± 10.611 to 214.94 ± 6.77, *T* = 413.5, *p* ≤ 0.001; RI 7: 509.78 ± 21.11 to 197.71 ± 6.45, *T* = 234, *p* ≤ 0.001; each Mann–Whitney *U* test). We observed an almost twofold increase in neuronal activity of serotonergic DRN neurons in *ePet-Cre/Cacna1a*^−/−^ mice on RI 7 compared with RI 3, while the DRN activity from control *ePet-Cre/Cacna1a*^+/+^ mice was not altered. These data suggest that enhanced DRN activity may lead to aggressive behavior as seen in our *ePet-Cre/Cacna1a*^−/−^ KO mice.

### Neuronal activity in VHMvl is increased in aggressive *ePetCre-Cacna1a*^−/−^ mice

Previous studies demonstrated that activity in the VHMvl mediates aggression-seeking behaviors (Falkner et al., 2016), and disinhibition of its glutamatergic cells results in increased aggression in mice (Lin et al., 2011; Lee et al., 2014; Hashikawa et al., 2017). Our laboratory has also previously shown that *c-fos* induction in the VHMvl is correlated with aggressive behavior in a serotonergic specific RGS2-overexpressing mouse model (Mark et al., 2019). To investigate whether neuronal activity is augmented in the VHMvl of our *ePetCre/Cacna1a*^−/−^ mice, we monitored *c-fos* levels in the VHMvl before and after RI tests. We analyzed our *ePet-Cre/Cacna1a*^−/−^*/tdTomato* and control *ePet-Cre/tdTomato* mice presented in Figure 5*A* also for their *c-fos* expression in the VHMvl glutamatergic neurons (eAAC1, red) (Fig. 5*C*). As expected, we observed 3.2 × more *c-fos* in *ePet-Cre/Cacna1a*^−/−^*/tdTomato* compared with control mice (363.667 ± 42.916 vs 113.333 ± 44.175, *t*_(4)_ = −4.065, *p* = 0.015, *t* test) after RI 7 (Fig. 5*D*). Not surprisingly, we found significantly more active neurons in our Ca_v_2.1-deficient mice after 3 and 7 d of the RI test (RI 3: *ePetCre-Cacna1a*^−/−^: 113.41 ± 2.2 to *Cacna1a*^+/+^: 55.17 ± 2.4, *t*_(22)_ = −17.870, *p* ≤ 0.001, *t* test; RI 7: *ePetCre-Cacna1a*^−/−^: 122.83 ± 7.45 to *Cacna1a*^+/+^: 85.58 ± 5.56, *t*_(22)_ = −13.866, *p* ≤ 0.001, *t* test) compared with control litter mice (Fig. 5*D*). We observed a 1.55-fold increase of *c-fos* expressing cells in the VHMvl of control mice from 662 on RI 3 to 1027 on RI 7 (*p* ≤ 0.001), while the *ePet-Cre/Cacna1a*^−/−^ mice showed a 1.8-fold increase in VHMvl active cells between RI 0 and RI 3, showing their reduced aggression threshold. Together, the observed increases in neuronal activity in both DRN and VHMvl in *ePetCre-Cacna1a*^−/−^ mice correlate with rises in aggression levels.

### Viral tracing reveals monosynaptic projections from the DRN to VHMvl in *ePet-Cre/Cacna1a*^−/−^ mice

**Figure 6.**
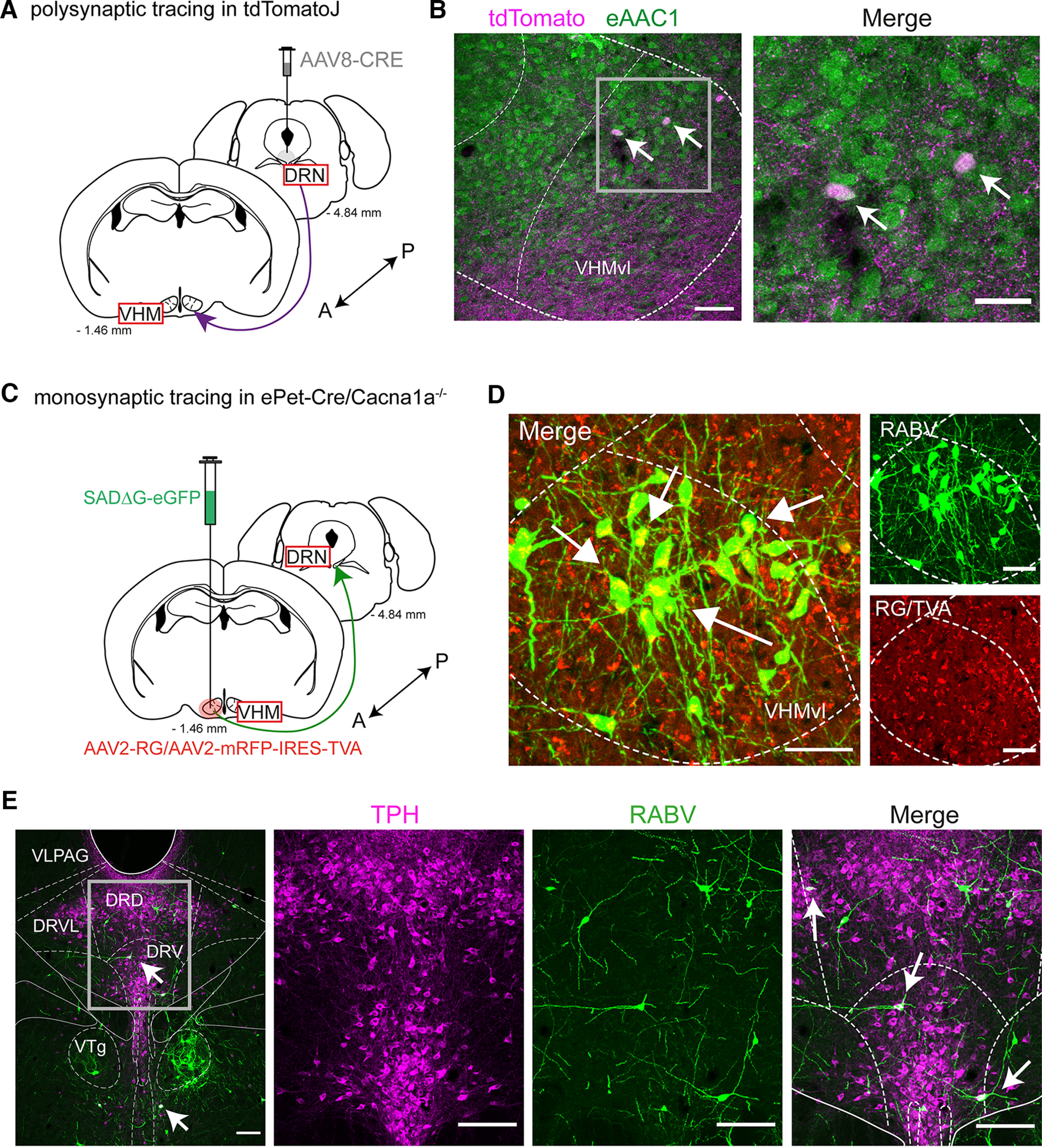
The VHMvl receives serotonergic input from the DRN in *ePet-Cre/Cacna1a*^−/−^ mice. A polysynaptic tracing approach (***A***) was conducted in *tdTomato* mice (*n* = 2). AAV8-Cre was injected in the DRN and analyzed for tdTomato^+^ axons in the VHMvl. ***B***, Example images depicting tdTomato^+^ axons and neurons in the VHMvl, which colocalized with the glutamate transporter (eAAC1) in glutamatergic cells (green). Scale bar, 30 µm. ***C***, To verify a monosynaptic projection between the DRN and VHMvl, a deletion-mutant rabies virus, SADΔG-eGFP, was injected into the VHMvl of *ePet-Cre/Cacna1a*^−/−^ and *Cacna1a*^+/+^ mice (*n* = 6). The virus can cross one synapse retrogradely, thus showing monosynaptic input regions to the injected area. ***D***, Example images of the injection site (VHMvl) of an *ePet-Cre/Cacna1a*^−/−^ mouse. Red represents the helper viruses AAV8-RG/AAV8-IRES-TVA providing the required glycoprotein for the membrane synthesis of rabies (green) and TVA receptor, which allows the RABV to enter the infected neuron. Double-fluorescent neurons (white arrows) indicate “starter neurons,” which receive input from other brain areas and can be identified by the expression of eGFP. Scale bars, 50 µm. ***E***, Transverse section of the DRN of the same mouse, where serotonergic neurons were identified by TPH antibody staining (magenta). Monosynaptic input neurons from the DRN to the VHMvl (white arrows) were identified by expression of eGFP. VLPAG, Ventrolateral periaqueductal gray; VTg, ventral tegmental nucleus. Scale bars, 150 µm.

In addition to the serotonergic system, the VHMvl is key player in aggressive behavior and generation of attacks (Hashikawa et al., 2016, 2017). Studies have shown that Esr^+^ neurons of the VHMvl project to the median raphe nucleus (Lo et al., 2019); however, a direct projection pathway from the DRN to the VHMvl has not been identified yet. We used a previously published polysynaptic, anterograde AAV8-WGA-Cre tracer injected into the DRN of tdTomato mice (Bohne et al., 2019). After 4 weeks of expression, we found tdTomato-positive projections from the DRN synapse on glutamatergic VHMvl cells, which were identified by the glutamatergic specific marker, glutamate transporter (eAAC1) (Fig. 6*B*). In addition, a strong tdTomato labeling of neurites within the VHMvl and adjacent areas was evident. To support these findings, we injected rabies monosynaptic tracer, SADΔG-eGFP (Niedworok et al., 2012; Bohne et al., 2019) into the VHMvl of *ePet-Cre/Cacna1a*^−/−^ mice (Fig. 6*C*). After 1 week, a robust expression of the modified rabies virus as indicated by the eGFP^+^ neurons in the VHMvl was demonstrated (Fig. 6*D*). We identified input neurons located in the dorsal and ventral parts of the DRN, which were retrogradely labeled by monosynaptic transport of the rabies virus (Fig. 6*E*). Interestingly, we did not observe significant innervation differences between our aggressive Ca_V_2.1 depletion mice and their littermate controls (data not shown, analysis of 6 control mice). Together, these data indicate that the DRN sends serotonergic projections directly to the VHMvl, thereby probably mediating enhanced neural activity and thus aggressive behavior in mice.

### Chemogenetic silencing of DRN serotonergic projections reduces neural activity in VHMvl and aggressive behavior in *ePetCre-Cacna1a*^−/−^ mice

**Figure 7.**
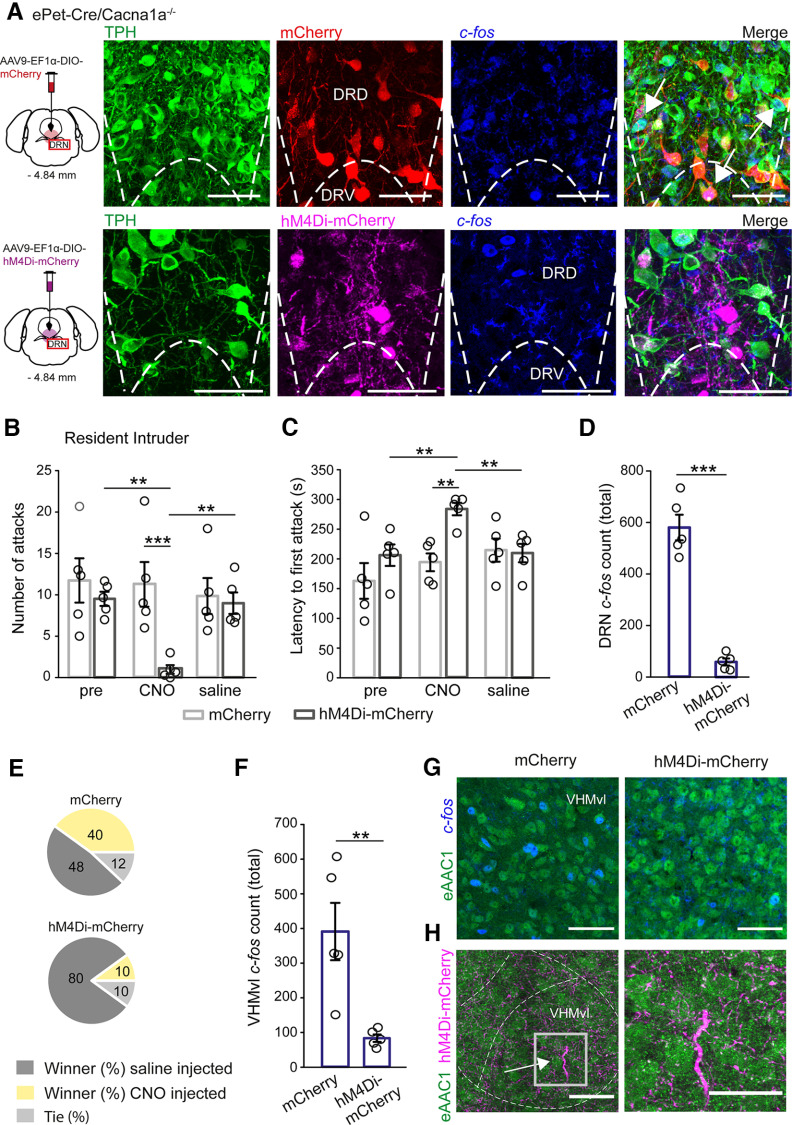
Injection of the inhibitory hM4Di-mCherry DREADD, but not control mCherry virus in the DRN of male *ePet-Cre/Cacna1a*^−/−^ mice rescues aggressive behavior. ***A***, Either AAV9-EF1α-DIO-mCherry (red) or AAV9-EF1α-DIO-hM4Di-mCherry (magenta) was injected into the DRN of each 5 *ePet-Cre/Cacna1a*^−/−^ mice and allowed to express for 4 weeks. Scale bars, 50 µm. ***B***, *ePet-Cre/Cacna1a*^−/−^ mice injected with the inhibitory DREADD showed 9.527 ± 0.856 attacks after 8 d of RI compared with 11.734 ± 2.68 attacks in control mice (*p* = 0.423). All mice were injected with 5 mg/kg CNO for 3 subsequent days 30 min before the RI test, and attacks were significantly reduced in hM4Di-mCh-injected mice compared with mCh-injected controls (0.993 ± 0.529 vs 11.266 ± 2.696, *p* = 0.001). Aggressive behavior was recovered in DREADD-injected mice following saline injections for 3 d (post) with a mean of 8.999 ± 1.299 attacks (*p* = 0.007). ***C***, Injection of CNO induced a delay in the latency to first attack in hM4Di-mCh-injected *ePet-Cre/Cacna1a*^−/−^ mice (206.199 ± 18.057 to 283.865 ± 10.536 s, *p* = 0.009) compared with control mice (162.998 ± 30.068 to 194 ± 14.982 s, *p* = 0.325) (*p* = 0.003). Subsequent saline injection recovered the latency to first attack in DREADD-injected mice to 209.732 ± 15.516 s (*p* = 0.008), while the latency for mCh control mice remained unaltered (214.733 ± 19.471, *p* = 0.370). ***D***, Analysis of *c-fos* expression in TPH^+^/virus^+^ neurons revealed CNO-mediated silencing of serotonergic neurons in the DRN of hM4Di-mCh-injected mice compared with control mCh-injected mice (58.4 ± 13.5 vs 580.4 ± 49.73, *p* ≤ 0.001). ***E***, *ePet-Cre/Cacna1a*^−/−^ DREADD-injected mice display increased submissive behavior compared with mCh-injected mice when intraperitoneally injected with CNO (10% vs 40% won fights) when competing against saline-injected mice of the same group (80% vs 48% won fights). ***F***, *c-fos* expression in glutamatergic neurons in the VHMvl shows that neuronal activity is reduced in DREADD-injected compared with mCh-injected mice when given CNO (83.6 ± 10.792 vs 391.2 ± 82.686, *p* = 0.008). ***G***, Example images *c-fos* (blue) overlaying with eAAC1 staining (green) for glutamatergic neurons in the VHMvl of both hM4Di-mCherry- and mCherry-injected mice. Scale bars, 50 µm. ***H***, hM4Di-mCherry^+^ axons were found in the VHMvl, strengthening projections from the DRN to the hypothalamic nuclei. Scale bars: overview, 50 µm; section, 30 µm. Data are mean ± SEM. Statistical significance was evaluated with a two-way repeated-measures ANOVA for comparison of two different groups of animals used for 2 or more different conditions (***B***) virus × treatment: df = 2, *F* = 4.745, *p* = 0.024, two-way repeated-measures ANOVA, (***C***) virus × treatment: df = 2, F = 4.538, *p* = 0.027, *t* test (***D***), and Mann–Whitney *U* test (***F***) (*p < 0.05; ***p < 0.001). See also Table 2.

In a previous study, Wong et al. (2016) showed that increased aggression was regulated by excitation of glutamatergic neurons in the VHMvl, since the lesioning of GABAergic inputs from the lateral septum onto glutamatergic cells in the VHMvl resulted in an increased aggression in mice. In this study, we found that aggression in *ePet-Cre/Cacna1a*^−/−^ mice is enhanced by deletion of Ca_v_2.1 in the serotonergic DRN neurons, which results in increased serotonergic firing rates and overall neural activity of the DRN and VHMvl, most likely in its glutamatergic neurons (see Fig. 5). To investigate whether serotonergic projections from the DRN to the VHMvl are directly mediating aggression, we used a chemogenetic approach either injecting serotonergic neurons in the DRN with an inhibitory DREADD (AAV9-EF1α-DIO-hM4Di-mCherry) or control mCherry virus (AAV9-EF1α-DIO-mCherry) in male *ePet-Cre/Cacna1a*^−/−^ mice (Fig. 7*A*) and monitored their aggressive behavior. We found robust expression of both mCherry and inhibitory DREADD in TPH^+^ neurons of the DRN (Fig. 7*A*), as well as their serotonergic projections in the VHMvl (Fig. 7*H*). Both groups (*n* = 5) developed stable aggressive behavior, displaying 11.734 ± 2.68 (mCh) and 9.527 ± 0.856 (hM4Di-mCh) attacks for 3 d (Fig. 7*B*) and comparable latencies to first attack (162.998 ± 30.068 s (mCh) vs 206.199 ± 18.057 s (hM4Di-mCh), *p* = 0.125) before CNO injection (Fig. 7*C*). After CNO injection, the number of attacks was only reduced in hM4Di-mCh-injected *ePet-Cre/Cacna1a*^−/−^ mice to 0.993 ± 0.529 attacks, while attacks in the control group remained stable at 11.266 ± 2.696 (*p* = 0.001; virus × treatment: df = 2, *F* = 4.745, *p* = 0.024, two-way repeated-measures ANOVA), thus decreasing their aggressive behavior to that of their WT littermates (see Fig. 3, pre vs CNO hM4Di-mCherry injected mice: *p* = 0.006). Injection of CNO had no effect on the latency to first attack in mCh-injected mice (162.998 ± 30.068 s vs 194.265 ± 14.982 s, *p* = 0.325), while the latency was significantly increased in hM4Di-mCh-injected mice (206.199 ± 18.057 s vs 283.865 ± 10.536 s, *p* = 0.009; virus × treatment: df = 2, *F* = 4.538, *p* = 0.027, two-way repeated-measures ANOVA), delaying onset of attacks by 46.12% compared with control mice (*p* = 0.003) (Fig. 7*C*). Subsequent saline injections starting 24 h later recovered the number of attacks in DREADD-injected mice (8.999 ± 1.299 attacks, *p* = 0.007) while simultaneously reducing the latency to first attack to pretreatment levels (209.732 ± 15.516 s, *p* = 0.008). Control mice displayed 9.859 ± 2.169 attacks with a latency of 214.733 ± 19.471 s when saline was injected. Quantitative analysis of *c-fos* expression (Fig. 7*A*) showed that neuronal activity was significantly reduced in serotonergic DRN neurons after CNO injection in DREADD-injected compared with mCh-injected mice (58.4 ± 13.5 vs 580.4 ± 49.73, *t*_(8)_ = 10.13, *p* ≤ 0.001, *t* test) (Fig. 7*D*). Moreover, silencing the serotonergic neurons in *ePet-Cre/Cacna1a*^−/−^ mice with the inhibitory DREADD enhanced submissive behavior in the tube displacement test, while mCherry injected mice were not affected (Fig. 7*E*). Thus, *ePet-Cre/Cacna1a*^−/−^ mice injected with CNO only won 10% of their battles against vehicle-injected mice, where vehicle-injected mice won 80% of their battles (10% were ties); 48% of vehicle-injected control mice won their battles against CNO injected mice (40%), with 12% observed ties. To verify that glutamatergic neurons in the VHMvl are influenced by serotonergic projections, we analyzed expression of *c-fos*, too. We found that *c-fos* expression in glutamatergic cells was significantly reduced in DREADD-injected *ePet-Cre/Cacna1a*^−/−^ mice compared with mCh-injected mice (391.2 ± 82.686 vs 83.6 ± 10.792, *T* = 40, *p*(est) = 0.012 *p*(exact) = 0.008, Mann–Whitney *U* test) (Fig. 7*F*,*G*), likely via hM4Di-mCh-positive neurites coming from the DRN (Fig. 7*H*; Table 2).

**Table 2.** Impact of CNO injection on motor behavior and locomotion in ePet-Cre/Cacna1a^−/−^ mice injected with AAV9-EF1a-DIO-mCherry or AAV9-EF1a-DIO-hM4Di_(Gi/o)_-mCherry in the DRN*^a^*

	ePet-Cre/Cacna1a^−/−^ (AAV9-EF1α-DIO-mch)			ePet-Cre/Cacna1a^−/−^ (AAV9-EF1α-DIO-hM4Di_(Gi/o)_-mch)		
Parameter	NaCl	CNO	*p*	NaCl	CNO	*p*
Open field						
Duration (s)						
Center	160.299 ± 12.944	124.745 ± 21.797	0.198	179.886 ± 7.17	84.493 ± 15.269	≤0.001
Intermediate	250.565 ± 262.649	272.831 ± 285.114	0.548	248.965 ± 11.708	195.378 ± 29.901	0.134
Border	491.936 ± 31.083	515.735 ± 47.175	0.685	461.916 ± 17.492	428.069 ± 47.037	0.151
Total distance (cm)	6680.046 ± 765.632	8848.778 ± 1393.271	0.210	10.503 ± 0.755	7.809 ± 0.605	0.505
Velocity (cm/s)	11.656 ± 17.753	10.14 ± 1.645	0.482	10.503 ± 0.755	7.809 ± 0.605	0.024
Rotarod						
Time (s)	109.42 ± 17.753	121.32 ± 12.369	0.597	115.873 ± 18.457	123.24 ± 14.36	0.761
Speed (rpm)	16.64 ± 2.153	18.8 ± 1.947	0.478	17.533 ± 2.101	18.32 ± 1.782	0.782

*^a^*Data are mean ± SEM.

Collectively, our data suggest that the voltage-gated PQ/type calcium channel is involved in regulating aggressive behavior in mice, likely via regulating the firing of serotonergic neurons in the DRN and present a new therapeutic target to treat aggressive and violent behavior.

## Discussion

In the present study, we report that deletion of Ca_V_2.1 from serotonergic neurons in the DRN results in increased aggressive behavior in the RI and tube displacement test (Fig. 3), enhanced firing rates of putative serotonergic neurons (Fig. 4) and increasing *c-fos* expression in the DRN and VHMvl (Fig. 5) in male *ePet-Cre/Cacna1a*^−/−^ compared with *Cacna1a*^+/+^ mice, while anxiety-like behavior is not affected. There is much evidence implicating a role of the serotonergic system in aggression. However, there are no reports implicating the Ca_v_2.1 role in DRN-mediated male aggression. Here we show that the Ca_v_2.1, primarily located at the presynapse and soma of neurons (Westenbroek et al., 1995), is required for regular synaptic transmission to maintain docile behavior.

**Figure 8.**
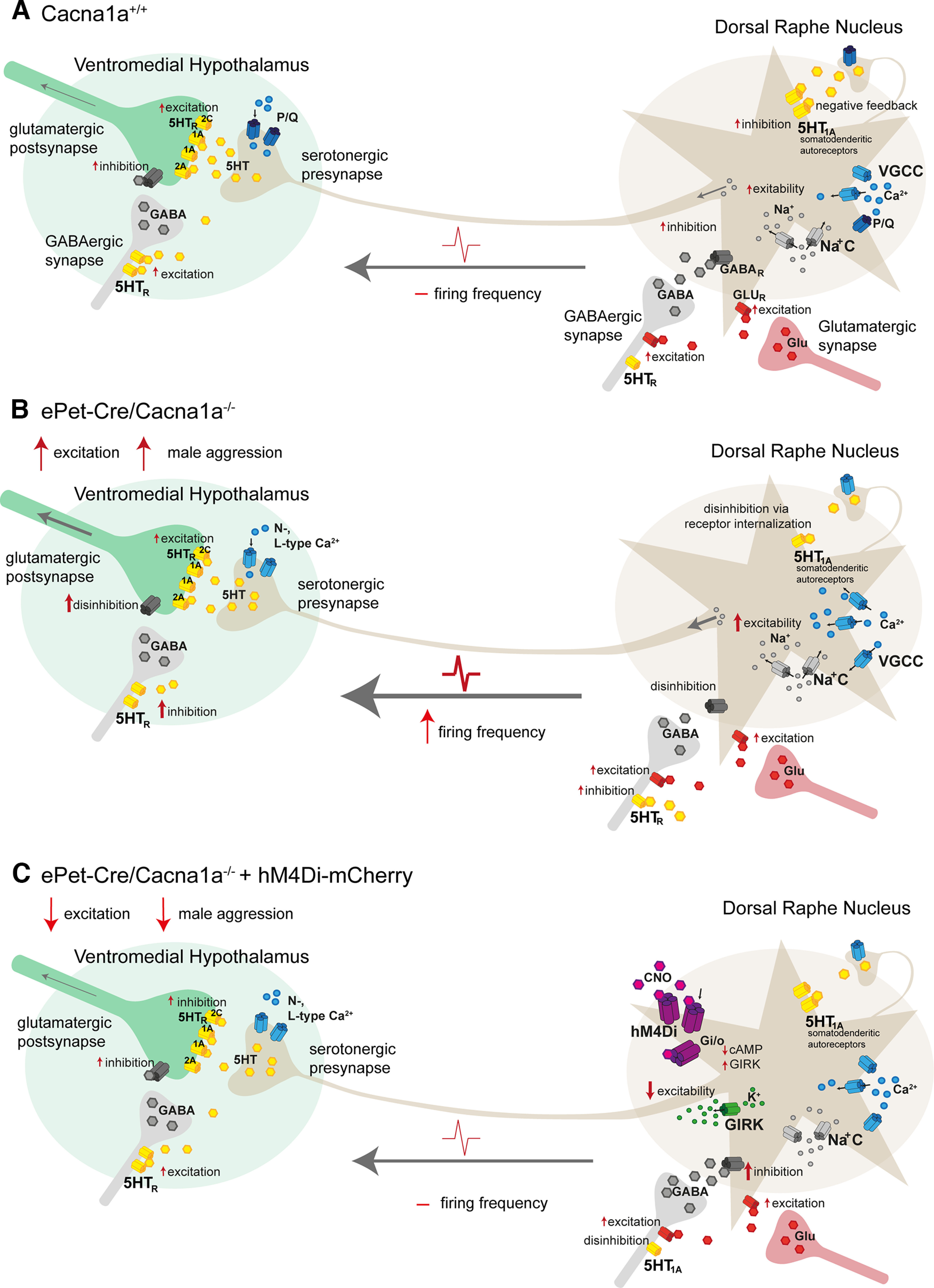
Schematic of how Ca_v_2.1 modulation of serotonergic synaptic transmission leads to male aggression in mice. ***A***, Control *Cacna1a*^+/+^ mice display regular serotonergic firing frequencies and serotonin release at the presynapse in the VHMvl. To ensure normal social behavior, voltage-gated calcium channels (VGCC), including L-, N-, T-, and P/Q-type channels mediating regular neuronal excitability, are kept in balance by adjacent GABAergic interneurons and somatodendritic 5-HT_1A_ autoreceptors, which reduce excitability. ***B***, *ePet-Cre/Cacna1a*^−/−^ mice display increased firing frequencies of serotonergic neurons in the DRN. Since the Ca_v_2.1, the main calcium channel mediating intrinsic pacemaker activity, is selectively deleted from neurons in the DRN, other VGCCs likely overcompensate for the loss, which results in increased firing of serotonergic neurons but decreased 5-HT release at its synapses in the VHMvl, likely leading to disinhibition of adjacent GABAergic interneurons and reduced 5-HT_R_ activation at the postsynapse, thus leading to increased aggressive behavior. This mechanism may be strengthened by 5-HT_1A_ autoreceptor internalization on DRN serotonergic neurons after their chronical and continuous activation, leading to disinhibition of the negative feedback mechanisms, further strengthened by unaltered glutamatergic inputs, supporting the increased firing rates in *ePet-Cre-Cacna1A*^−/−^ mice. ***C***, When the inhibitory DREADD receptor, AAV9-hM4Di_(Gi/o)_-mCherry, is expressed on DRN serotonergic neurons in male *ePet-Cre/Cacna1a*^−/−^ mice, a decrease in male aggressive behavior is observed. The introduction of the inhibitory GPCR and subsequent activation via CNO application terminates in a reduction of cytosolic cAMP levels and enhanced activation of GIRK channels, leading to K^+^ efflux and reduced excitability, thus normalizing serotonergic cell firing and presumably restoring 5-HT release at the presynapse to control *Cacna1a*^+/+^ levels. GABA release from DRN interneurons mediates additional effective inhibition of DRN neurons, resulting in decreased firing and limited excitation of postsynaptic glutamatergic neurons. Additionally, GABAergic interneurons in the VHMvl inhibit the glutamatergic neurons, thus helping to decrease VHMvl neuronal activity and subsequent male aggression in *ePet-Cre/Cacna1a*^−/−^ mice.

Ca_v_2.1 expression in the DRN could not be confirmed unequivocally (Penington and Fox, 1995) until recently, where an mRNA expression study verified its expression on DRN neurons (Templin et al., 2012). Immunohistochemical staining of the Ca_V_2.1 protein in our control *Cacna1a*^+/+^ and *ePet-Cre/Cacna1a*^−/−^ mice verified Ca_v_2.1 expression on serotonergic neurons in the DRN (Fig. 1). We hypothesize that the deficiency of the Ca_v_2.1 on serotonergic neurons in the DRN most likely results in impaired synaptic transmission to connected brain regions (e.g., the VHMvl; Fig. 8) since the calcium channel is strongly coupled to the exocytosis machinery (Qian and Noebels, 2001; Li et al., 2007). Several studies showed that 5-HT deficiencies or imbalances lead to altered aggressive behaviors in mice (Chiavegatto et al., 2001; Osipova et al., 2009; Angoa-Pérez et al., 2012; Mosienko et al., 2012; Audero et al., 2013), confirming their role in aggression. Our *ePet-Cre/Cacna1a*^−/−^ mice display increased firing rates of putative serotonergic neurons identified by their unique firing properties (Fig. 4), possibly constituting compensatory coping mechanisms to balance out decreased 5-HT release. These mice display increased aggressive behavior toward male intruders (Fig. 3), and neuronal activity in the DRN is significantly increased compared with control mice (Fig. 5). Despite the enhanced serotonergic activity during aggressive behavior, additional local feedforward and feedback circuits, including GABAergic, glutamatergic, and other inputs, are likely involved, too (Adell et al., 2002). Although not further investigated in our study, various studies explored the influence of different neurotransmitters on DRN neuronal activity and behavioral outcome. For example, the excitatory neurotransmitter glutamate was shown to increase in the DRN during aggressive behavior (Takahashi et al., 2015). Additionally, serotonergic and GABAergic neurons in the DRN receive both almost identical excitatory and inhibitory inputs from, for example, the lateral hypothalamus (Weissbourd et al., 2014; Zhou et al., 2017). GABAergic neurons were shown to regulate DRN serotonergic activity (Challis et al., 2013), implicating that a precise and context-dependent neuromodulation of serotonergic neurons via GABAergic and glutamatergic inputs mediates aggressive to docile behavior. Although not explored, it is possible that GABAergic synaptic transmission is impaired or that the strong local inhibitory feedback mechanisms via 5-HT_1A_ autoreceptors on cell bodies (Courtney and Ford, 2016; Albert and Vahid-Ansari, 2019) and 5-HT_1B_ on axon terminals (McDevitt and Neumaier, 2011) may be internalized or desensitized because of chronic activation via 5-HT in our *ePet-Cre/Cacna1a*^−/−^ mice, further contributing to the enhanced firing rates (Fig. 3).

Despite the DRN, the VHMvl emerged as an essential region for controlling the initiation of attacks (Falkner et al., 2014; Yang et al., 2017) and ∼95% of the neurons within the VHMvl are excitatory glutamatergic cells (Choi et al., 2005), forming strong synaptic contacts with each other (Nishizuka and Pfaff, 1989). Various studies in mice using optogenetic or chemogenetic activation of VHMvl neurons coupled its increased neuronal activity to the initiation of attacks, even toward female mice or lifeless objects (Lin et al., 2011; Lee et al., 2014; Falkner et al., 2016; Yang et al., 2017) and VHMvl activity rises during or even before attacks (Falkner et al., 2014). We thus explored the neuronal connection from the DRN to the VHMvl by use of a deletion-mutant rabies virus, SADΔG-eGFP (Niedworok et al., 2012) in our *ePe-Cre/Cacna1a*^−/−^ and *Cacna1a*^+/+^ mice and found direct monosynaptic projections from the DRN to the VHMvl (Fig. 6), which have not been reported before (Waselus et al., 2011; Biagioni et al., 2016; Nordman and Li, 2020). Additionally, based on *c-fos* counts in the VHMvl, we observed that its activity rises after the RI test in our Ca_V_2.1-deficient *ePet-Cre/Cacna1a*^−/−^, but not control Cacna1a^+/+^ mice (Fig. 5), suggesting direct neuromodulatory mechanism from the DRN to the VHMvl. In accordance with the serotonergic deficiency theory, we postulate that the loss of the Ca_V_2.1 channel from serotonergic DRN presynapses in *ePet-Cre/Cacna1a*^−/−^ mice terminates in decreased 5-HT release onto glutamatergic neurons and GABAergic interneurons in the VHMvl, leading to disinhibition and increased aggressive behavior (Fig. 8*B*). This idea is supported by our *c-fos* counts, showing that prolonged RI tests result in enhanced neuronal activity in both DRN and VHMvl (Fig. 5). Since neurons in the VHMvl predominantly express inhibitory G_i/o_-coupled 5-HT_1A_ receptors (Wright et al., 1995), despite fewer excitatory G_q_-coupled 5-HT_2A_ and 5-HT_2C_ receptors (Pompeiano et al., 1994; Li et al., 1997; Clemett et al., 2000; da Silva et al., 2011), a prevailing inhibitory effect of 5-HT on the glutamatergic cells in the VHMvl can be assumed (Fig. 8*A*). The hypothesis of VHMvl disinhibition is further supported by another study, where artificial overexpression of 5-HT_1A_ receptors or the suppression of serotonergic firing in the DRN resulted in increased aggressive behavior in male mice during the RI test (Audero et al., 2013).

Since the VHMvl has such strong excitatory properties, neighboring brain regions, such as lateral hypothalamus, juxtraventromedial region, ventral zone, tuberal nucleus (Canteras et al., 1994), the medial nucleus of the amygdala (Canteras et al., 1995), the bed nuclei of the stria terminalis (Dong and Swanson, 2004), lateral septum (Risold and Swanson, 1997), and medial preoptic area (Simerly and Swanson, 1988), provide strong local inhibitory control of VHMvl activity via GABAergic interneurons, which are also stimulated via 5-HT release from the DRN. The importance of GABAergic interneurons in controlling aggressive behavior was shown by injection of a GABA_A_ agonist into the VHMvl of male mice, resulting in reduced poke rates during a self-initiated aggression task (Falkner et al., 2016). For our *ePet-Cre/Cacna1a*^−/−^ mice, we postulate a disinhibitory effect of both glutamatergic VHMvl neurons and inbound GABAergic synapses caused by reduced 5-HT release of the DRN presynapse (Fig. 8*B*), leading to increased activation of the glutamatergic neurons represented by increased aggressive behavior (Fig. 3) and enhanced *c-fos* expression in our *ePet-Cre/Cacna1a*^−/−^ mice (Fig. 5). This mechanism is supported another study, where the local microinjection of the GABA_B_ receptor agonist baclofen into the DRN resulted in enhanced aggressive behavior in mice (Takahashi et al., 2010), possibly because increased GABA release inhibits serotonergic neurons, which reduces 5-HT release at the synaptic cleft at glutamatergic neurons in the VHMvl and finally resulting in disinhibition of these neurons and aggressive behavior.

Since potential serotonergic cell firing frequencies were enhanced in *ePet-Cre/Cacna1a*^−/−^ mice (Fig. 4), we aimed to normalize their firing rates by injecting the inhibitory DREADD receptor (hM4Di-mch) into the DRN (Fig. 7). Interestingly, we found that chemogenetic inhibition of serotonergic hM4Di-mCh-infected neurons in the DRN via CNO injection rescued docile behavior in our *ePet-Cre/Cacna1a*^−/−^ mice in the RI and tube displacement test, while aggressive behavior in control mCh virus-injected mice was not altered after CNO administration (Fig. 7*B*,*C*). The DREADD receptor activation via CNO stimulates GIRK channels, leading to reduced intracellular cAMP levels and therefore reduced excitability (Fig. 8*C*). To date, no study investigated the effects of chemogenetic silencing of DRN serotonergic neurons in aggression; however, our *c-fos* data support the idea that CNO-induced inhibition of serotonergic DRN neurons normalizes their firing rates and directly correlates with decreased activity in the VHMvl (Fig. 7).

Together, we demonstrate that deletion of the Ca_v_2.1 from serotonergic neurons in the DRN terminates in enhanced firing rates and increased aggressive behavior, supported by increased expression of the immediate early gene marker *c-fos* in DRN and VHMvl, a node essential for initiation and expression of attacks. We verified monosynaptic projections from the DRN to the VHMvl and postulate that Ca_v_2.1 depletion causes reduced synaptic serotonergic transmission to neurons in the VHMvl, leading to a decrease in GABAergic interneuron inhibition of glutamatergic neurons, and thus overexcitation of VHMvl glutamatergic neurons and aggressive behavior. Expression of the inhibitory DREADD receptor in DRN neurons restores docile behavior in *ePet-Cre/Cacna1a*^−/−^ mice, possibly by normalizing DRN firing rates based on *c-fos* counts, thus restoring adequate 5-HT release at the VHMvl synaptic cleft, activating GABAergic interneurons and inhibiting glutamatergic VHMvl neurons.
